# Supplementary material for: Patterns of Intron Gain and Loss in Fungi
Source: PLoS Biol. 2004 Nov 30;2(12):e422. doi: 10.1371/journal.pbio.0020422 (PMC532390; doi:10.1371/journal.pbio.0020422)
Supplement: Table S1 — Also available at http://genes.mit.edu/NielsenEtAl/. (4.3 MB ZIP). [file pbio.0020422.st001.zip › NielsenEtAl/html/315.html]

AN4490.1.NCU02943.1.MG04219.1.FG09359.1


```
 CLUSTAL W (1.82) Multiple Sequence Alignments - Introns Inserted


Sequence 1: NCU02943.1	570 aa
Sequence 2: MG04219.1	526 aa
Sequence 3: FG09359.1	525 aa
Sequence 4: AN4490.1	532 aa
Alignment Length: 583 aa
Number Identitical Residues: 172 aa
Alignment Score (without introns) 9383


MG04219.1 	-----------------------MLSRLAI~RTLRAPALQGLG-RTTAARQTTSLLAART
NCU02943.1	MTTCPPLSLTLNSVCGAIGAGNKNSQNIKV1MMLSRAAVRSIAGARVAAHAASPLLSQRT
FG09359.1 	-------------------MLSRLAQASRL~GLMTARLSQPVASPSLRAIPATAPHFASP
AN4490.1  	-----------------MLRRAILPLTRPS~GLVSAPRLSALPVSHSRCYAKGSKPKTPY
          	                                 :       :      .    .      

MG04219.1 	----WTRGMAKNNK------PAPTPSQQTS-KSNAPSEEQASSPAPKQSENATK~PEASD
NCU02943.1	LPAVWTRSMAKDNKPPKFSKPESTPAQKATPKAPEPAESEKAEQKQQQQQQQQT~PAESE
FG09359.1 	----WLRSYSKRSSGQPPKESKKKPSQAQN-DAEAAKTPEKPAENDVNKASEQS~PEAPK
AN4490.1  	----KLPESVKSSKPEQPAKPSQQEQYAAEQAEFETTSDPQANTANTTSQASSS0PESSP
          	          * ..     ..              .     .      .    . *  . 

MG04219.1 	AQPEQVPFN--LPDLTKGLPSTFEYEASG--------ADKASKMALEGVAS---------
NCU02943.1	PEPEIDLSK--LPDLRGGIPTTLEYEMAQKEAGKKPVAGEEAETQAEGAEGPEAATSGSG
FG09359.1 	EGEQIPFHK--LPDLTQGIPSTLFEEMGGDKK-----KEQQALQELEEAES---------
AN4490.1  	SQSEQDAPQRPLPDLTQGIPSTLAAELEARSKK----SGSGTLNLTEDPSR-----FEED
          	   :    : .****  *:*:*:  *    .        . :    *           ..

MG04219.1 	AGGRGKGELPASAYVSSSDRRRQKVAQWVFYGFVAGGLFGVVFLGRNWEDEEERAKHADV
NCU02943.1	GGGRKKGQLPDSAYVSSTEKRRQKMANWAFIAAGLALVGGTIYLGREWD-EEELEKHHDI
FG09359.1 	----KGNERDRSEYVSTSERNRKWWTRFMLTAVAAGGTLSLLYMGRNWEDTIEAERHSDS
AN4490.1  	YSDDGRGDIPKGGYESSLDRKRARMAKLMYALFLLGSVGGMAYLGRNWDTVEEENAHPDV
          	 ..   .:   . * *: ::.*   :.        .   .  ::**:*:   *   * * 

MG04219.1 	PDGWTPVLWWKRAMARMGDTVSYYQEPAFEKLLPDPDPVNSPPYTLCISLEDLLVHSEWT
NCU02943.1	PNGWGLGLWWKRAKARMTGTVSYYQEPAFEKLLPDPDPSFERPYTLCISLEDMLVHSEWT
FG09359.1 	PNGPSPSLWWKRAKARMTESVTYYQEPAFEKLLPDPDPTFERPYTLCLSLDDLLIHSEWT
AN4490.1  	PSGWSFGLWYNRIKARMGDFTSYYKDPAFPKLLPDEDPNLRQPYTLVLSLEDLLVHSEWS
          	*.*    **::*  ***   .:**::*** ***** **    **** :**:*:*:****:

MG04219.1 	RDHGWRVAKRPGVDYFIRYLSQYYELVLFTSVPYGIAEPLWRKMDPFRFVQWPLFREATK
NCU02943.1	RDHGWRLAKRPGVDYFLRYLSQYYEIVLFTSVPFANAEPIVRKMDPYRFIMWPLFREATK
FG09359.1 	REHGWRIAKRPGVDYFIRYLSQYYELVLFTTTPYATGEPVMRKLDPFRLILWPLYREATK
AN4490.1  	REHGWRVAKRPGVDYFLRYLNQYYELVLFTSVPSMMADQVLRKLDPYRIIRWPLFREATR
          	*:****:*********:***.****:****:.*   .: : **:**:*:: ***:****:

MG04219.1 	YVDGKIVK0DLSYLNRDLSKVIIIDTNPEHVSAQPENAIILPKWTGDAQDKDLVALIPFL
NCU02943.1	YKDGEIVK0DLSYLNRDLSKVIIIDTDPKHVRAQPENAIVLPKWKGDPKDTELVSLVPFL
FG09359.1 	FEDGEIVK0DLSYLNRDLSKVIIIDTKAKHVRNQPDNAIILDPWKGDKDDKNLVNLIPFL
AN4490.1  	YKDGEYIK0DLSYLNRDLSKVILIDTKEEHARLQPENAIILDKWNGNPKDKTLVALIPFL
          	: **: :* *************:***. :*.  **:***:*  *.*: .*. ** *:***

MG04219.1 	EYIHTMQYPDVRKVLKSFEGKNISEEFARREAIARKKFQEQLEQNRKKHPNKPGAGVFGA
NCU02943.1	EFIHTMNFPDVRKVLKSFEGQHIPTEFARREAIARAEHNKLVAAKAKKAG-------LGS
FG09359.1 	EYIHTMQYSDVRKVIKSFDGKDIPTEFARREAIARKEFQAKQLTHKHKHGSG-----VGA
AN4490.1  	EYLAGMGVDDVRTVLKSFEGQSIPIEFAKREKAMRERFEKELAEEQKKRPRSG----MGS
          	*::  *   ***.*:***:*: *. ***:**   * ..:     . :*         .*:

MG04219.1 	LGNAFGLKPSKMSMMVPVEGEESVADALAQGKMLQDIARERGQRQYERIDKMVRENGEKW
NCU02943.1	LGARFGIKPSKLNPMA-MEGEEDPSEAFAKGKMIQDIARERGMRNYLAMEEEIKKNGEMW
FG09359.1 	LGNMLGLKPSNMNMMVSPDGEQNPAEAFAQGKMLQDVARERGQRNYMELEKQIRENGEKW
AN4490.1  	LASALGLKSSAR----TLDGEQLPSAGLQEGKMLWDQIRERGQKNYELIEKEIRENGEKW
          	*.  :*:*.*      . :**:  : .: :***: *  **** ::*  ::: :::*** *

MG04219.1 	LKEEAEMMEKMQAEAMANMKTGFMGWFG---SSEQKDGAKDEPKKA
NCU02943.1	LKMEQEAQEKAQKEMMKNMQSSVFGWFGGAPSGEQQSGESE--KKA
FG09359.1 	LKEEAAMMEAAQKEAMNSMMGSFGGWFG---------GNNPPEKKA
AN4490.1  	LAEMAAEEEKLRQEQMESMKGSLTGFFG--------GGKKE-----
          	*       *  : * * .*  .. *:**        .* .
```
